# Adsorption Behavior of Methylene Blue Dye by Novel CrossLinked *O*-CM-Chitosan Hydrogel in Aqueous Solution: Kinetics, Isotherm and Thermodynamics

**DOI:** 10.3390/polym13213659

**Published:** 2021-10-24

**Authors:** Nouf Faisal Alharby, Ruwayda S. Almutairi, Nadia A. Mohamed

**Affiliations:** 1 Department of Chemistry, College of Science, Qassim University, P.O. Box 6644, Buraydah 51452, Saudi Arabia; r.almotery@qu.edu.sa (R.S.A.); NA.AHMED@qu.edu.sa (N.A.M.); 2 Department of Chemistry, Faculty of Science, Cairo University, Giza 12613, Egypt

**Keywords:** carboxymethyl chitosan hydrogel, MB dye, adsorption kinetics, adsorption isotherms, adsorption thermodynamic

## Abstract

The chemical cross-linking of carboxymethyl chitosan (*O*-CM-chitosan), as a method for its modification, was performed using trimellitic anhydride isothiocyanate to obtain novel cross-linked *O*-CM-chitosan hydrogel. Its structure was proven using FTIR, XRD and SEM. Its adsorption capacity for the removal of Methylene Blue (MB) dye from aqueous solution was studied. The effects of different factors on the adsorption process, such as the pH, temperature and concentration of the dye, in addition to applications of the kinetic studies of the adsorption process, adsorption isotherm and thermodynamic parameters, were studied. It was found that the amount of adsorbed MB dye increases with increasing temperature. A significant increase was obtained in the adsorption capacities and removal percentage of MB dye with increasing pH values. An increase in the initial dye concentration increases the adsorption capacities, and decreases the removal percentage. It was found that the pseudo-second-order mechanism is predominant, and the overall rate of the dye adsorption process appears to be controlled by more than one step. The Langmuir model showed high applicability for the adsorption of MB dye onto *O*-CM-chitosan hydrogel. The value of the activation energy (*E_a_*) is 27.15 kJ mol^−1^ and the thermodynamic parameters were evaluated. The regeneration and reuse of the investigated adsorbent was investigated.

## 1. Introduction

Water contamination with dyes is a major problem worldwide, and a serious public environmental problem. Dyes are used in a wide range of applications such as in the textile, chemical, paper, paint, leather, food and coating industries. When a very small amount of dye is released in water, an obvious color is observed, which greatly influences the water quality and prevents light and oxygen from reaching into water. The accumulation of dyes in water poses a tremendous threat to the ecological system due to the high toxicity of these dyes for human health and aquatic living organisms [1,2]. These dyes are carcinogenic, mutagenic or teratogenic, causing a high level of damage to the genetic materials. Some dyes are related to splenic sacromas, cyanosis, shock, quadriplegia, bladder cancer, hepatocarcinoma, dermatitis, allergy and skin irritation [3]. The harmful effect of the dyes is associated with their non-biodegradability, resistance and stability under various conditions [1].

The dyes’ removal from wastewater effluents is highly necessary, particularly before they are discharged into the environment. Various treatment technologies are used in the removal of dyes, such as coagulating/flocculating, electrocoagulation/electroflotation, separation (via ion exchanging and membranes) and ozonation [4]. There are some restrictions on the application of these technologies, such as high-energy consumption, the high cost of the used chemicals, production of toxic by-products and their poor performance at low dye concentrations [5,6,7,8].

The adsorption process is the most efficient technology for dyes’ removal from wastewater. This is due to its low cost, simplicity, ease of operation, and high efficiency in the removal of a large variety of pollutants, as well as the abundance of a wide range of adsorbents that are easily restored for reuse [1,2,3,4,5,6,7,8,9,10]. Although the activated carbon is efficient in dye absorption, it has an increased cost and is difficult to regain and dispose [11]. The use of several adsorbents suitable for the removal of low concentrations of dyes, including activated carbon, silica, chitin and peat, is reported. However, the adsorption capacity of these adsorbents is not high [12]. Thus, the use of low-cost and highly efficient adsorbents is still under development and has attracted much interest in recent decades [4].

Carboxymethyl chitosan (CM-chitosan) is an amphoteric ether derivative of chitosan and possesses active hydroxyl (–OH), carboxylic (–COOH), and amino (–NH_2_) groups in its repeating units [13]. It has a gel-forming ability, high viscosity, low toxicity, hydrophilicity, anti-bacterial properties, and good biocompatibility and biodegradability. It also shows a high adsorption capacity towards various pollutants, such as dyes and heavy metals [14]. However, it is a water-soluble derivative in a wide pH range due to the possession of both amino and carboxylic groups in its structure. The solubility of CM-chitosan in acidic media limits its use in adsorbing dyes from wastewater because the effluents are usually acidic. Thus, the chemical modification of CM-chitosan via its basic –NH_2_ groups is a good way to lower its solubility at low pH values and increase both its chemical stability and its mechanical strength [15]. Moreover, the introduction of additional carboxyl groups to CM-chitosan increases its adsorption capacity for the removal of cationic dyes [16].

In the present study, *O*-CM-chitosan was chemically cross-linked using trimellitic anhydride isothiocyanate, to develop a stable and durable biopolymeric material and incorporate additional carboxylic groups as active anionic sites, to bind Methylene blue dye as a cationic dye from its aqueous solutions. The study focused on the impact of different parameters on the adsorption process including pH, temperature and initial dye concentrations. In addition, the kinetic, isotherms, and thermodynamics of the adsorption process were studied to find the MB dye adsorption mechanism.

## 2. Materials and Methods

**Figure 1:**
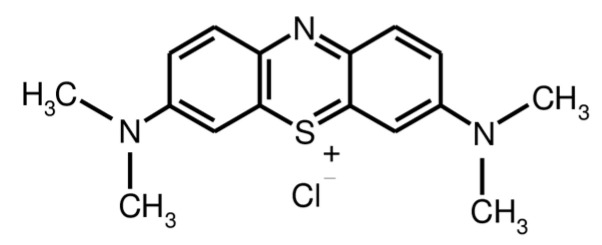
Chemical structure of MB dye.

Chitosan (with a deacetylation degree of 88% and a molecular weight of 1.0–3.0 × 10^5^ g mol^−1^) and monochloroaceic acid were purchased from Across Organics (Fair Lawn, NJ, USA). Trimellitic anhydride chloride and Methylene Blue (MB) dye (Figure 1) were obtained from Sigma-Aldrich (Munich, Germany). The buffer powder pillows of pH (4, 7 and 9) were supplied by Hach Company (Loveland, CO, USA). The other chemicals and reagents were provided by Loba chemie PVT. Ltd, (Bombai, India), were of analytical grade and were used as-received.

### 2.1. Preparation of O-CM-Chitosan

Chitosan (10 g) was magnetically stirred in 200 mL aqueous sodium hydroxide solution (20% *w/v*) at room temperature for 15 min. Monochloroacetic acid (30 g) was slowly added to the solution and stirred at 40 °C for two h. Aqueous acetic acid solution (10% *v*/*v*) was added until the reaction medium was completely neutralized; then, an excess of aqueous methanol solution (70% *v*/*v*) was added. The product (*O*-CM-chitosan) was obtained by filtration, washed repeatedly with methanol, and then dried at 60 °C to constant weight (15 g) [17]. The degree of substitution of the resulting *O*-CM-chitosan was 0.75, determined using the previously reported method [18].

### 2.2. Synthesis of O-CM-Chitosan Hydrogel

**Scheme 1:**
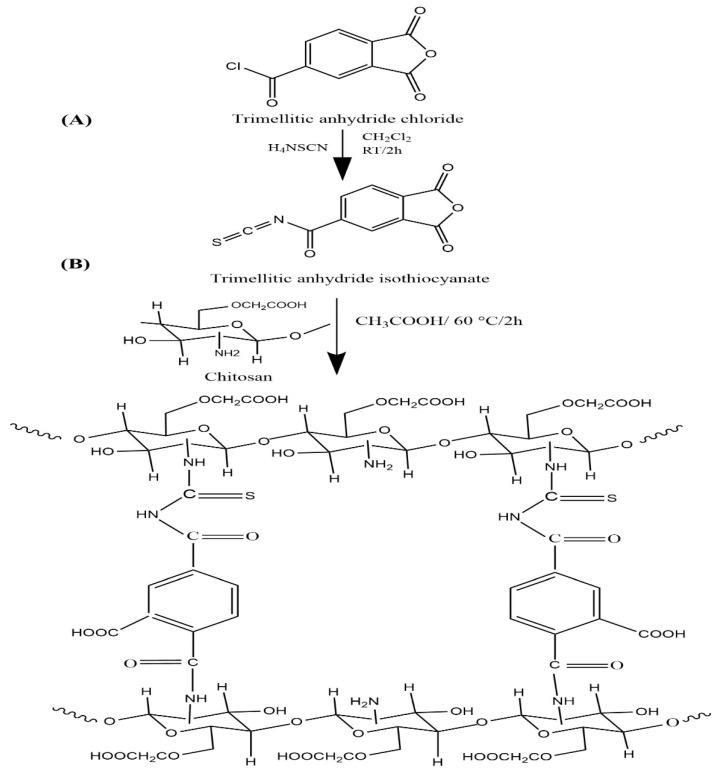
Preparation of *O*-CM-chitosan hydrogel.

The *O*-CM-chitosan hydrogel was prepared following the method reported in our previous work [19]. Ammonium thiocyanate (1 g, 13.14 mmol) was stirred in methylene chloride (20 mL). Then, solid trimellitic anhydride chloride (2.77 g, 13.14 mmol) was slowly added, followed by 1 mL of polyethylene glycol-400 as a phase transfer catalyst. The reaction admixture was agitated on a magnetic stirrer at room temperature for 2 h. The white precipitate (ammonium chloride) produced by this reaction was isolated by filtration to obtain trimellitic anhydride isothiocyanate (Scheme 1A) as a filtrate. This filtrate was slowly added to *O*-CM-chitosan (6.65 g, 26.28 mmol), dissolved in 216 mL aqueous acetic acid solution (1% *v*/*v*), and agitated using a mechanical stirrer at 60 °C for two h, then at 25 °C for another twelve h. The resulting homogenous *O*-CM-chitosan hydrogel (Scheme 1B) was precipitated via treatment with a sodium carbonate solution until it reached pH 7 to remove the acetic acid medium. The obtained yellowish white product was filtered, soaked in methanol for 24 h to dewater and desalt, filtered again and finally dried at 60 °C.

### 2.3. Measurements

#### 2.3.1. FTIR Spectrometry

FTIR spectrometer Nicolet 6700 (Thermo Scientific, Waltham, MA, USA) was employed to measure the FTIR spectra of the chitosan, O-CM-chitosan and O-CM-chitosan hydrogel. Each sample was thoroughly mixed with KBr and then compressed under a hydraulic pressure of 400 kg cm^−2^ to make a pellet. The spectra were recorded at wave numbers ranging from 4000 to 500 cm^−1^, at 25 °C.

#### 2.3.2. X-ray Diffractometry

X-ray diffraction patterns of chitosan, O-CM-chitosan and O-CM-chitosan hydrogel were recorded by X-ray diffractometer (Rigaku Ultima-IV). The powder of each sample was loaded on the sample holder and scanned in the reflection mode at a 2θ angle over a range from 5° to 90° at a speed of 8° min^−1^.

#### 2.3.3. Scanning Electron Microscopy

The topography of chitosan, O-CM-chitosan and O-CM-chitosan hydrogel was photographed using the high-resolution Field emission-scanning electron microscope (JSM-7610F). The powder of each sample was mounted on the surface of the SEM specimen holder and coated with a thin layer of gold before photographing at an accelerated voltage of 15 kV and a magnification of 2000×.

#### 2.3.4. Solubility

The soluble portion of the sample was estimated by agitating 0.05 g of the sample in 10 mL of various diluted acids (AcOH, HCl and HNO_3_, 1% *v*/*v*) at 25 °C for 24 h. The sample residue was completely dried and then weighed. The soluble portion can be calculated using Equation (1) [20]

% Soluble portion = [(W_0_ − W_1_)/W_0_] × 100 (1)

where W_0_ is the initial weight of the sample and W_1_ is the dried weight of the sample residue.

#### 2.3.5. Swell Ability

The swelling capacity of chitosan, O-CM-chitosan and O-CM-chitosan hydrogel samples in different media was estimated by immersing 0.02 g of each dry sample in 20 mL of various buffer solutions (pH 4, pH 7 and pH 9) at different temperatures (25 °C, 35 °C and 55 °C) without disturbance overnight. The swollen sample was removed from the immersion media, swiftly swabbed using a filter paper to remove the droplets on its surfaces and reweighed. Its swelling capacity can be determined using Equation (2) [21]

% Swelling capacity = [(W_1_ − W_0_)/W_0_] × 100 (2)

where W_0_ and W_1_ are the weights of the dry and swelled samples, respectively. Swelling measurements were performed many times and the average of three comparable results were taken.

### 2.4. Adsorption of MB Dye onto O-CM-Chitosan Hydrogel

The adsorption of MB dye from its aqueous solution onto *O*-CM-chitosan hydrogel was performed using batch experiments. Throughout each experiment, a known amount of adsorbent (0.05 g) was mixed with a dye solution (50 mL, 1000 mg L^−1^). The mixture was shaken using a water bath shaker set at 80 rpm, and the temperature was controlled by a thermostat until equilibrium. The concentration of the non-adsorbed dye was determined by measuring its absorbance at 664 nm using a Shimadzu UV/Vis 1601 spectrophotometer, Japan. The adsorption was also studied at varied pH values of 4, 7 and 9, while keeping other conditions constant (50 mL of dye solution (1000 mg L^−1^), 0.05 g of adsorbent at 25 °C). HCl or NaOH solution (0.1 M) was used to adjust the pH value of the dye solution. The adsorption was studied at different temperatures of 25, 35, 45 and 55 °C at pH 7. The adsorption was investigated using different concentrations of dye (400–1000 mg L^−1^) at room temperature and pH 7.

**Figure 2:**
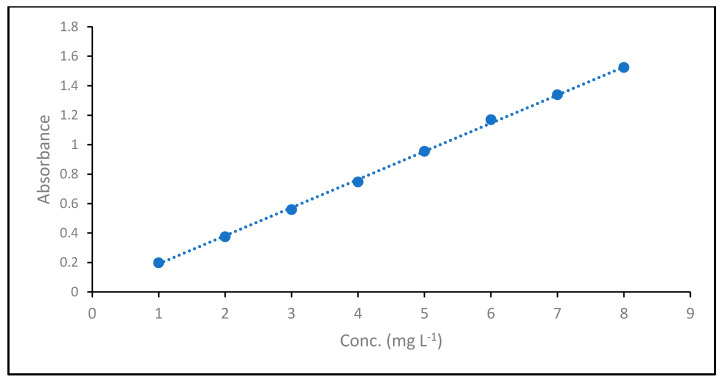
Absorption Coefficient of MB dye.

To determine the unknown dye concentrations, a calibration curve was established between the dye concentrations and absorbance, as shown in Figure 2, and the absorbance coefficient was determined using Beer–Lambert law (Equation (3))

A = εlc(3)

where A is the absorbance, ε is the molar absorptivity (L mol^−1^ cm^−1^), l is the path length of the cuvette containing the sample (cm) and c is the concentration of dye in solution (mg L^−1^). The obtained absorbance coefficient was 0.191.

Then, the amount of the dye on the adsorbent and the adsorption capacity can be calculated using Equation (4) and Equation (5), respectively,

(4) $$q_{t}=\frac{\left(C_{o}-C_{t}\right)\;V}{W}$$

(5) $$q_{e}=\frac{\left(C_{o}-C_{e}\right)\;V}{W}\;$$

where *q_t_* and *q_e_* (mg g^−1^) are the amount of dye on the adsorbent at certain time t and at equilibrium time, respectively. *C_o_* (mg L^−1^) is the initial concentration of dye, *C_t_* (mg L^−1^) is the concentration of dye at time t, *C_e_* (mg L^−1^) is the concentration of dye at equilibrium time, *V* is the volume of the dye solution (L) and *W* is the weight of the adsorbent (g).

The removal efficiency of dye can be calculated from Equation (6) [8]

(6) $$\%\;\mathrm{Removal}\;\mathrm{efficiency}=\left(\frac{\left(C_{o}-C_{e}\right)}{C_{o}}\right)\times 100$$

where *C_o_* and *C_e_* (mg L^−1^) are the initial concentration and equilibrium concentration of the dye in solution, respectively.

#### 2.4.1. Kinetic Studies

The kinetics of MB dye adsorption onto *O*-CM-chitosan hydrogel were analyzed by four kinetic models: pseudo-first-order, pseudo-second order, intraparticle diffusion and Elovich models.

Pseudo-First-Order Model

The linearized form of the pseudo-first-order model under initial and final boundary conditions (*t* = 0 to *t* = *t* and *q_t_* = 0 to *q_t_* = *q_t_*) can be expressed by Equation (7)

(7) $$\log\left(q_{e}-q_{t}\right)=\log q_{e}-\frac{k_{1}}{2.303}t$$

where *q_e_* is the capacity of adsorption at time of equilibrium (mg g^−1^), *q_t_* is the capacity of adsorption at time *t* (mg g^−1^), k_1_ (min^−1^) is the pseudo-first-order rate constant. *log* (*q_e_* − *q_t_*) was plotted versus *t*, giving a linear relation by which the values of *q_e_* and *k*_1_ can be estimated from the intercept and the linear slope, respectively.

Pseudo-Second-Order Model

The linearized form of pseudo-second-order model under the initial and final boundary conditions (*t* = 0 to *t* = *t* and *q_t_* = 0 to *q_t_* = *q_t_*) can be expressed by Equation (8)

(8) $$\frac{t}{q_{t}}=\frac{1}{k_{2}q_{e}^{2}}+\frac{t}{q_{e}}$$

where *q_e_* and *q_t_* (mg g^−1^) are the amount of dye adsorbed on the adsorbent at equilibrium and time *t*, respectively. *k*_2_ (g mg^−1^min^−1^) is the rate constant of the pseudo-second-order model. The linearized relationship is obtained by plotting *t*/*q* against *t*, as the values of *q_e_* and *k*_2_ can be obtained from the slope and intercept, respectively.

Intraparticle diffusion

This model can be represented by Equation (9)

*q_t_* = (*K_p_ t*^1/2^) + *C_i_* (9)

where *K_p_* (mg g^−1^min^−1/2^) is the intraparticle diffusion constant; *C_i_* os the boundary layer thickness. By plotting a linear relation between *q_t_* versus *t*^1/2^, the values of *k_p_* and *C_i_* can be obtained by slope and intercept calculations, respectively.

Elovich model

This kinetic model can be expressed by Equation (10)

(10) $$q_{t}\;=\left(\frac{1}{\beta }\right)\ln(\alpha \beta )+\frac{1}{\beta }\ln\;t$$

where α (mg g^−1^ min^−1^) is the initial rate constant of adsorption, β (g mg^−1^) is the constant of desorption associated with the surface coverage and the activation energy of chemical adsorption, and *q_t_* (mg g^−1^) is the amount of adsorbed dye at time *t* (min). Both α and β values can be obtained by plotting *q_t_* versus ln *t*. [22].

Kinetic Validity

The validation of the investigated kinetic models can be determined using the normalized standard deviation Δ*q_e_* (%), which can be calculated using Equation (11) [23]

(11) $$\Delta q_{e}(\%)=100\;\times \;\sqrt{{\left[\frac{\left(q_{e,exp}-q_{e,cal}\right)/q_{e,exp})}{n-1}\;\right]}^{2}}$$

where *q_e_*_,*exp*_ (mg g^−1^) is the experimental capacity of adsorption, *q_e_*_,*cal*_ (mg g^−1^) is the calculated capacity of adsorption for the above models and n is the number of datapoints.

#### 2.4.2. Adsorption Isotherm

The isotherm is a crucial consideration when designing an adsorption system [24]. The isotherm helps to show the adsorbate interaction with the adsorbent surface at a certain temperature. This isotherm shows the relationship between the concentrations of adsorbed solute and the concentration of the solute in solution [25]. By studying the isotherm in the present work, different concentrations of MB dye (500–1000 mg L^−1^) were used. Typically, 10 mL of dye solution was added to 0.01 g of *O*-CM-chitosan hydrogel at 25 °C and pH 7. The mixture was shaken in a water shaker with agitation speed 80 rpm. The concentration of un-adsorbed dyes was determined by measuring the absorbance of dye solution. The adsorption isotherm was analyzed by four isotherm adsorption models: Langmuir, Freundlich, Temkin and D-R.

Langmuir Isotherm Model

Langmuir isotherm is the most common adsorption isotherm. This isotherm is based on three main assumptions: (i) the formation of monolayer adsorbate coverage on the homogenous adsorbent surface; (ii) there is no interaction between the adsorbed molecules; (iii) the adsorbate molecules bind to the adsorption active sites and cannot migrate over the surface. Langmuir adsorption isotherm can be expressed by Equation (12) [22]

(12) $$\frac{C_{e}}{q_{e}}=\frac{1}{\left(q_{\max}.K_{L}\right)}+\frac{C_{e}}{\left(q_{\max}\right)}$$

where *q_e_* (mg g^−1^) is the amount of adsorbed dye at equilibrium, *C_e_* (mg L^−1^) is the equilibrium concentration of dye in solution, q_max_ (mg g^−1^) is the maximum monolayer coverage adsorption capacity, and K_L_ (L mg^−1^) is a Langmuir coefficient, related to adsorption energy. The q_max_ and K_L_ values can be estimated from the slope obtained by plotting *C_e_*/*q_e_* versus *C_e_*, resulting a linear relationship, since q_max_ and K_L_ can be determined from the slope 1/q_max_ and intercept 1/(q_max_ K_L_), respectively. An important characteristic of the Langmuir isotherm model can be expressed by the separation factor or equilibrium factor (R_L_) (Equation (13)).

(13) $$R_{L}=\frac{1}{\left(1+K_{L}C_{o}\right)\;}$$

where K_L_ is the Langmuir constant and C_o_ is the initial concentration of the adsorbate in solution. This value can be used to define the adsorption process: unfavorable (R_L_ > 1), linear (R_L_ = 1), favorable (0 < R_L_ < 1) and irreversible (R_L_ = 0) [26].

Freundlich isotherm model

The Freundlich isotherm model is based on the formation of multilayer adsorbate over a heterogenous adsorbent surface. In addition, the active sites possess different adsorption energies. The Freundlich model can be expressed by Equation (14) [27].

*q_e_* = *K_F_ C_e_*^1/n^ (14)

The linearized form of Freundlich isotherm model can be expressed by Equation (15).

(15) $$\mathrm{Ln}q_{e}=\ln K_{F}+\frac{1}{n}\ln C_{e}$$

where *q_e_* (mg g^−1^) is the adsorbed amount at equilibrium and *C_e_* (mg L^−1^) is the equilibrium concentration of the dye at a constant temperature. *K_F_* and n are empirical constants related to the capacity and intensity of adsorption, respectively. The values of the constant K_F_ and indictor 1/*n* are obtained by plotting ln *q_e_* against ln *C_e_*. These constants are utilized to define the favorability and feasibility of the adsorption process. Generally, if 0 < 1/*n* < 1 this represents a favorable adsorption process; 1/*n* = 0 indicates an irreversible adsorption and 1/*n* > 1 indicates unfavorable adsorption [28].

Temkin isotherm model

Temkin model is an important model that accounts for the factors that explain the interaction between the adsorbent and adsorbate. Temkin model suggests that (i) by completing the coverage, the adsorption heat decreases linearly, (ii) the binding energies are uniformly distributed. This model can be expressed by Equation (16)

(16) $$q_{e}=B_{T}\ln K_{T}+B_{T}\ln C_{e}$$

where *B_T_* (J mol^−1^) is the Temkin constant that is controlled by temperature, *K_T_* (Lg^−1^) is the binding constant of Temkin isotherm, *q_e_* (mg g^−1^) is the amount of dye adsorbed at equilibrium and *C_e_* (mg L^−1^) is the dye equilibrium concentration at a constant temperature. The values of *B* and *K*_*T*_ constants were obtained from the slope and intercept achieved by plotting *q_e_* against ln *C_e_* [22].

Dubinin–Radushkevich isotherm

To differentiate between the chemisorption and physisorption, the Dubinin–Radushkevich isotherm can be applied. This isotherm can explain the formation of multilayers onto the microporous adsorbents. This does not assume a homogenous surface, so it is more general than Langmuir isotherm. However, it can be used to describe the heterogeneity of the adsorbent surface at low coverage. The D-R isotherm can be expressed by Equation (17)

ln *q_e_* = ln (*q_m_*) − βε^2^ (17)

where *q_m_* (mg g^−1^) is the monolayer saturation capacity, β (mol^2^ kJ^−2^) is the activity coefficient, which is related to the mean free energy of adsorption per dye mole when transferred from infinity in the solution to the adsorbent surface. ε is the Polanyi potential, which can be determined by Equation (18)

(18) $$\varepsilon =RT\;\ln\;(1+\frac{1}{C_{e}}\;)\;$$

where *R* (8.314 J mol^−1^K^−1^) is the gas constant and *T* (K) is the absolute temperature.

The β and ln *q_m_* values in Equation (17) can be obtained from the intercept and the slope obtained from a plot between ln *q_e_* versus ε^2^. The mean free energy can be determined by Equation (19).

(19) $$E=(\;\frac{1}{{\left(2\beta \right)}^{0.5}}\;)$$

If the value of the mean free energy equals 8 < E < 16 kJ mol^−1^, then the process is chemisorption; if it is less than 8 kJ mol^−1^, the process is physisorption [29].

#### 2.4.3. Thermodynamic Studies

Studying thermodynamics accounts for the direction and feasibility of the adsorption process. By studying the thermodynamics, an important parameter can be obtained; changes in Gibbs free energy, Δ*G*° (kJ mol^−1^), enthalpy change, Δ*H*° (kJ mol^−1^) and entropy change, Δ*S*° (J mol^−1^ K^−1^) were calculated for the studied dye absorption using different temperature degrees of 25, 35, 45 and 55 °C according to Equations (20) and (21) [23]

Δ*G*° = −*RT*ln *K*_*c*_ (20)

(21) $$\ln\;k_{c}=\frac{\Delta S{}^{\circ}}{R}-\frac{\Delta H{}^{\circ}}{RT}$$

where *R* (8.314 J mol^−1^ K^−1^) is the universal gas constant, *K_c_* (*q_e_*/*C_e_*) is the distribution coefficient and *T* (K) is the absolute temperature. The thermodynamic parameters can be calculated by plotting ln *K_c_* versus 1/*T*.

#### 2.4.4. Determination of Adsorption Activation Energy

The impact of temperature on the adsorption of MB dye onto *O*-CM-chitosan hydrogel was investigated previously. The rate constant of pseudo-second-order, *k*_2_, was used to estimate the activation energy using the Arrhenius equation, as shown in Equation (22) [30].

(22) $$\ln k_{2}=\ln A-\frac{E_{a}}{RT}$$

where *E_a_* is the activation energy (J mol^−1^), A is the Arrhenius frequency factor, *R* is the molar gas constant (8.314 J mol^−1^ K^−1^) and T is the absolute temperature (K). *E_a_* is determined by plotting ln *k*_2_ versus inverse temperature (1/*T*). The slope gives the value of *E_a_*.

### 2.5. Regeneration Studies

The regeneration studies for *O*-CM-chitosan hydrogel were carried out by washing the adsorbent using distilled water to get rid of the non-adsorbed dye. The hydrogel was immersed in 20 mL of ethanol as a desorption medium at pH 7 and 25 °C. The amount of desorbed dye was calculated using Equation (23)

(23) $$\%\;\mathrm{Dye}\;\mathrm{desorption}=\frac{q_{d}}{q_{a}}\times 100$$

where *q_d_* is the amount of MB dye desorbed from the dye-saturated adsorbent (mg g^−1^) and *q_a_* is the amount of the dye adsorbed onto the adsorbent (mg g^−1^) [31].

## 3. Results and Discussion

### 3.1. Synthesis of the O-CM-Chitosan Hydrogel

Recently, we prepared four cross-linked chitosan hydrogels using trimellitic anhydride isothiocyanate as a cross-linker. The resulting chitosan hydrogels were found to be more hydrophilic, more swellable, more resistant to microbes and more adsorptive of Congo red and basic red 12 dyes in comparison with chitosan [19].

Thus, to increase these characteristics, our goal was to cross link the more functionalized *O*-CM-chitosan using the same cross-linker. Scheme 1A illustrated the preparation of a trimellitic anhydride isothiocyanate cross-linker, in which the trimellitic anhydride chloride reacted with ammonium thiocyanate. Methylene chloride was used as a reaction medium and polyethylene glycol-400 was utilized as a phase transfer catalyst. The reaction was carried out at room temperature for a period of 120 min. Scheme 1B demonstrated the synthesis of a novel *O*-CM-chitosan hydrogel. The reaction proceeded between amino groups of the *O*-CM-chitosan and anhydride ring and the isothiocyanate linkage of trimellitic anhydride isothiocyanate.

#### 3.1.1. FTIR Characterization of the *O*-CM-Chitosan Hydrogel

**Figure 3:**
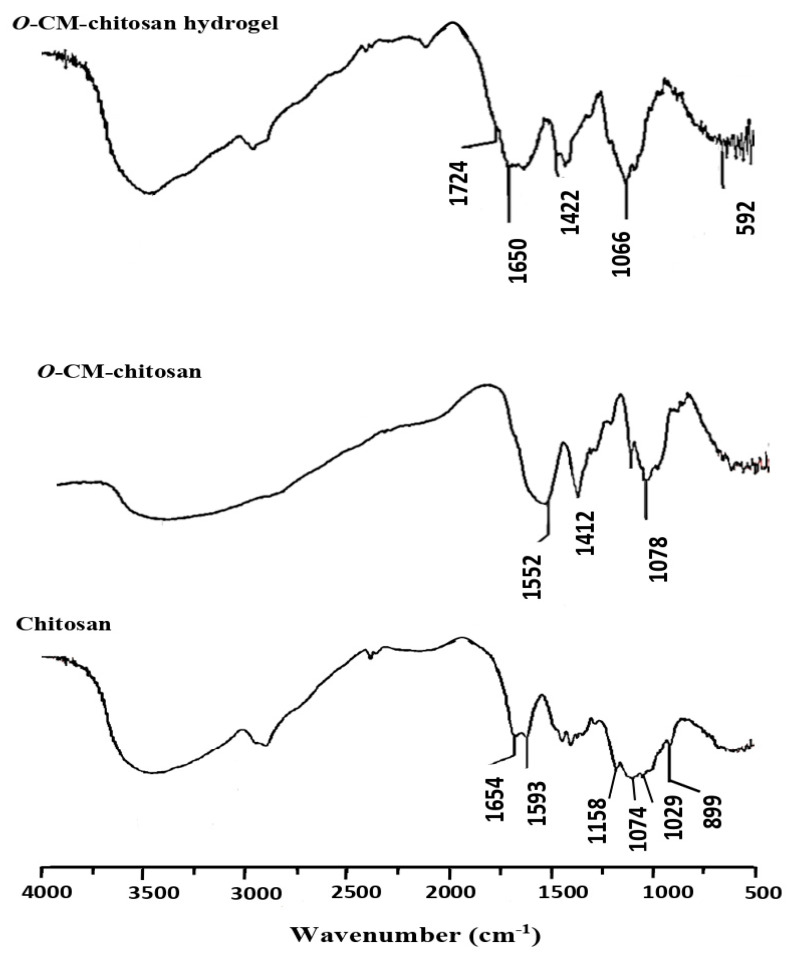
FTIR spectra of chitosan, *O*-CM-chitosan and *O*-CM-chitosan hydrogel.

Chitosan was characterized by four FTIR absorption peaks at 1158, 1074, 1029 and 899 cm^−1^, corresponding to its saccharide moiety (Figure 3). The hydroxyl and amino groups showed a strong, broad peak at 3600–3000 cm^−1^. There are two additional weak peaks at 1654 and 1593 cm^−1^, related to amide I and amide II, respectively, signalizing the high degree of deacetylation for chitosan. The amide I peak at 1654 cm^−1^ was overlapped with the deformation peak of the amino group at 1600 cm^−1^ to provide a strong peak [19].

On the other hand, *O*-CM-chitosan (Figure 3) showed similar bands to toset obtained in chitosan, in addition to a new band at 1412 cm^−1^ (strong), which was assigned to the symmetrical stretching vibration of the carboxylate group. The overlap between asymmetrical stretching vibrations of the carboxylate group at 1552 cm^−1^ and deforming vibrations of the NH_2_ group at 1600 cm^−1^ resulted in an intensive band. Further, the absorption band of C-O bond of the secondary hydroxyl group appeared to be more intensive and moved to the wavenumber 1078 cm^−1^. These results showed that the carboxymethylation reaction occurred on C6 [17].

Figure 3 also showed the FTIR spectrum for the *O*-CM-chitosan hydrogel, in which a new absorption band assigned to the –COOH group of the cross-linking linkage appeared at 1724 cm^−1^. The absorption band of –COOH group interfered with that of NH group. Another new absorption band appeared at 1650 cm^−1^ due to the overlapping bands of CONH, the NH of secondary amide and aromatic C=C bonds. The broad, strong absorption band at 1066 cm^−1^ is due to the existence of the C=S group overlapped with C-O absorption. The N-C-S group showed two absorption bands at 1422 and 592 cm^−1^ [19]. Thus, these data evidence the successful synthesis of the hydrogel (Scheme 1B).

#### 3.1.2. X-ray Diffraction of the *O*-CM-Chitosan Hydrogel

**Figure 4:**
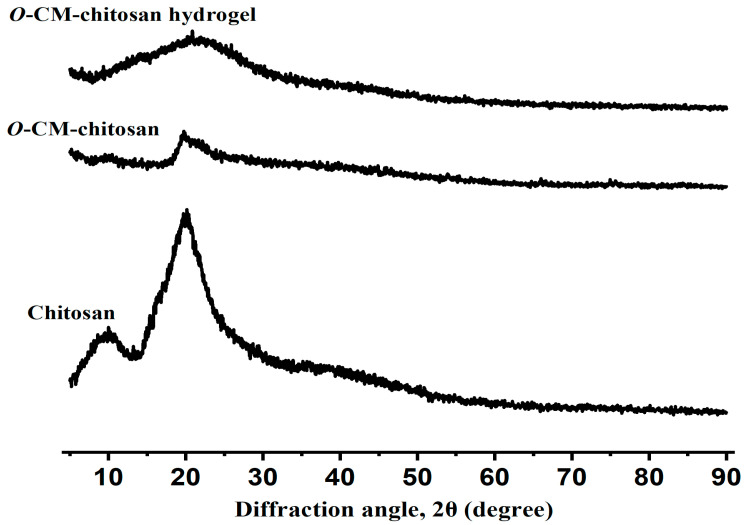
Powder X-ray diffraction of chitosan, *O*-CM-chitosan and *O*-CM-chitosan hydrogel.

The changes that occurred on the internal structure of the prepared hydrogel could be investigated using X-ray diffraction techniques. The X-ray diffraction pattern of chitosan (Figure 4) demonstrated two peaks, assigned to its amorphous and crystalline sections at the diffraction angles (2θ) 10° and 20°, respectively [32]. This can be ascribed to the existence of numerous OH and NH_2_ functional groups with high polarity, which allow for the formation of potent intra- and intermolecular hydrogen bonds. The X-ray diffractogram of *O*-CM-chitosan showed a peak at 2θ = 20°, characterized by a lower intensity as compared with the intensity of the same peak in virgin chitosan, referring to its decreased crystalline nature (Figure 4). After insertion of the long cross-linking linkages, the O-CM-chitosan hydrogel chains stayed away from each other and their hydrogen bonds were broken, decreasing the crystallinity of the hydrogel, and forming a highly amorphous structure with an accessible open matrix (Figure 4).

#### 3.1.3. Scanning Electron Microscopy (SEM) of the *O*-CM-Chitosan Hydrogel

**Figure 5:**
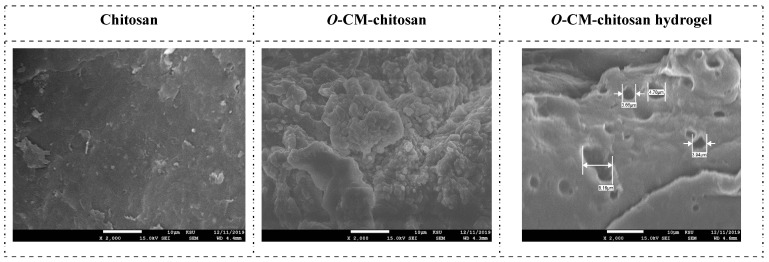
SEM micrographs of chitosan, *O*-CM-chitosan and the *O*-CM-chitosan hydrogel.

The smooth and polished nature of the topography of chitosan (Figure 5) was completely changed after the carboxymethylation process via the appearance of lumps on the surface of *O*-CM-chitosan. These resulted from the incorporation of carboxymethyl groups into chitosan. After the cross-linking process, the hydrogel topography showed a highly porous surface. These pores are presumed to act as water permeation zones and interaction centers of the hydrogel’s highly polar functional groups with exterior stimulants. The pore size can be ascribed to the length of the cross-linking linkages, permitting the absorption of a large quantity of liquids and supporting interactions between the hydrogel and incoming constituents. Therefore, the prepared hydrogel can be considered a promising candidate for some engineering applications, such as the absorption of dyes and metal ions.

#### 3.1.4. Solubility of the *O*-CM-Chitosan Hydrogel

Contrary to virgin chitosan and *O*-CM-chitosan, the prepared *O*-CM-chitosan hydrogel could not be dissolved in diluted acids (AcOH, HCl and HNO_3_ (1 % *v*/*v*)). This is due to the consumption of the *O*-CM-chitosan amino groups during the cross-linking process, which are responsible for their solubility in the diluted acid solutions.

#### 3.1.5. Swell Ability of the *O*-CM-Chitosan Hydrogel

Table 1 summarized data on the swelling capacity of chitosan, *O*-CM-chitosan and *O*-CM-chitosan hydrogel under different pH value and temperature conditions. Chitosan’s high sensitivity to the acid media of pH 4 is attributed to the abundance of -NH_2_ groups along its chains, which could easily be converted to NH_3_^+^ ions. The increment in these repelled mobile cations leads to osmotic imbalance because of their similar charges. This enhances liquid-spreading into the chitosan, resulting in an increment in its swelling capacity. Therefore, in acidic media, chitosan can serve as a polycationic material. Chitosan has a greater swelling capacity at pH 4 than at pH 7, which is greater than that at pH 9.

The *O*-CM-chitosan was found to be more swellable than chitosan at all the examined pH values. This is due to its greater hydrophilicity, resulting from the existence of -NH_2_ and –COOH functional groups along its chains. At pH 4, the -NH_2_ groups are converted to NH_3_^+^ ions (polycationic material), while at pH 9, the –COOH groups were deprotonated to carboxylate anions (–COO^−^, polyanionic material), increasing the number of mobile free ions and, consequently, osmosis inside the *O*-CM-chitosan material, and thus improving the quantity of the penetrated liquid. On the other hand, at pH 7, the –COOH groups can interact with the -NH_2_ groups, producing –COO^−^ and -NH_3_^+^ ions. Thus, the *O*-CM-chitosan behaves as a polyanionic and polycationic material (non-pH-dependent negative and positive charges for *O*-CM-chitosan). *O*-CM-chitosan has a greater swelling capacity at pH 9 than at pH 7, which is greater than that at pH 4.

The swelling capacity of the *O*-CM-chitosan hydrogel is also dependent on the tested pH values. This can be attributed to the existence of additional carboxylic groups, thiourea and amide linkages, in addition to the voids resulting from the cross-linking process. Therefore, it acts as a polyanionic and polycationic material, which is dependent on the pH value of the media. At pH 4, the protonation of NH, C=O and C=S groups to NH_2_^+^, C=OH^+^ and C=SH^+^ ions occurs, whereas the –COOH groups remain as they are (a polycationic hydrogel). Furthermore, in comparison to chitosan and *O*-CM-chitosan, the greater swelling capacity of the hydrogel can be ascribed to the voids included in hydrogel matrix, which contain H_2_O molecules. On the other hand, at pH 9, deprotonation of the –COOH groups to carboxylate anions occurs, whereas the functional NH, C=O and C=S groups are unaltered, producing a polyanionic hydrogel. In comparison to the parent *O*-CM-chitosan, the greater swelling capacity of the hydrogel in the alkaline media at pH 9 is due to the presence of an additional –COOH group (arising from the cross-linking process), as well as the voids included in the hydrogel matrix, which contain H_2_O molecules.

The *O*-CM-chitosan hydrogel exhibited a higher swelling percentage in alkaline medium than in neutral and acidic media. In an acidic medium, its hydrophilic groups (NH, C=O and C=S) are converted to NH_2_^+^, C=OH^+^ and C=SH^+^ ions, which repel each other, generating a repulsion force between the polymeric molecules. Moreover, the increment in the mobile ions inside the hydrogel matrix increases the osmotic pressure variance both outside and inside, which is the driving force for its swelling. The increment in the swelling capacity may also be ascribed to the free water content, which has a high mobility due to its low interaction with the polymer molecules. These impacts, and the capillary wetting of inter-connected open hydrogel pores, are believed to cause the higher swelling capacity in acidic media [33]. However, at pH 9, the –COOH groups turn to –COO^−^, and thus the swelling capacity can be ascribed to the difference in osmotic pressure and the repulsion interactions arising between –COO^−^ anions. Thus, the penetrated H_2_O molecules inside the hydrogel are bonded with –COO^−^ anions. The increment in the swelling capacity of the hydrogel at pH 9 compared to that at pH 7 is attributed to the ionization of –COOH to –COO^−^ carboxylate anions.

The swelling capacity of chitosan, *O*-CM-chitosan and *O*-CM-chitosan hydrogel was improved when temperature of the soaking media increased at all the examined pH values. This is due to improvements in the mobility of the generated ions and, consequently, their electrostatic interactions, increasing osmotic pressure inside the hydrogel and its swelling capacity.

### 3.2. Adsorption of MB Dye Using O-CM-Chitosan Hydrogel

#### 3.2.1. Adsorption Studies

A new *O*-CM-chitosan hydrogel was utilized for the removal of MB dye. Its adsorption capacity was studied by investigating the impact of different parameters, such as temperature, pH and initial dye concentration.

##### Effect of Temperature

**Figure 6:**
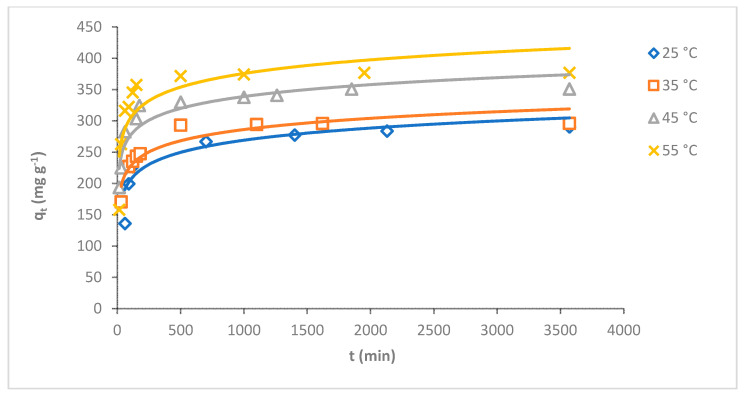
Effect of temperature on adsorption of MB dye onto *O*-CM-chitosan hydrogel at different time intervals (adsorbent mass 0.05 g, dye solution (50 mL, 1000 mg L^−1^) and pH 7).

The temperature is the main factor influencing the adsorption process [34]. The adsorption capacity of MB dye on *O*-CM-chitosan hydrogel was investigated at various temperatures 25, 35, 45 and 55 °C as a function of time (adsorbent mass 0.05 g, 50 mL of dye solution, with a concentration of 1000 mg L^−1^, and pH 7). The results are illustrated in Figure 6. It is obvious that the adsorption capacity of MB dye gradually decreased with increasing temperature. The adsorption capacity increased from 290.6 to 377.0 mg g^−1^ when temperature increased from 25 to 55 °C, respectively. The removal percentage increased gradually with increasing temperature, at 29.1, 29.6, 35.1 and 37.7% at temperatures of 25, 35, 45 and 55 °C, respectively.

The results suggested that the adsorption of MB dye onto the *O*-CM-chitosan hydrogel is an endothermic process in nature [35]. The high adsorption capacity of *O*-CM-chitosan hydrogel in dye removal might be due to the high swelling ability of hydrogel, which helps in the high diffusion of dye molecules in its porous structure. This allows for the penetration of dye molecules into the inner surface of hydrogel, due to the hydrophobic and electrostatic interactions between hydrogel and dye molecules [36]. Moreover, the *O*-CM-chitosan hydrogel is partly swollen with increasing temperatures, increasing the area of its internal structure, and resulting in the increased penetration of dye molecules into the internal layer of adsorbent and increased dye uptake [37]. This result is in good agreement with a previous study on the adsorption of MB onto *N*,*O*-CM-chitosan [38].

##### Impact of the pH of Dye Solution

**Figure 7:**
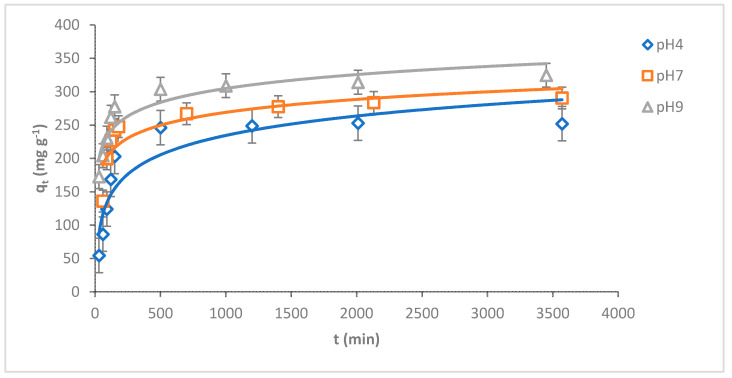
pH impact of MB dye adsorption onto *O*-CM-chitosan hydrogel at different time intervals (adsorbent dose 0.05 g, 50 mL of dye solution (1000 mg L^−1^), at 25 °C).

pH plays a key role in adsorbing adsorbates onto the adsorbent surface. The adsorption capacity of MB dye onto *O*-CM-chitosan hydrogel was investigated at various dye solution pH (4, 7 and 9) as a function of time (adsorbent amount 0.05 g, 50 mL of dye solution (1000 mg L^−1^) at 25 °C). The results are illustrated in Figure 7.

As can be seen from the Figure, there is a remarkable variation in the adsorption capacity of MB dye onto *O*-CM-chitosan hydrogel due to the changing pH values of the dye solution. The adsorption capacity increased from 252.9 to 324.6 mg g^−1^ with increasing pH from 4 to 9. Additionally, the removal percentage increased with increasing pH values, equaling 25.3, 29.1 and 32.5% at pH 4, 7 and 9, respectively.

At a high pH, the negative charge density on *O*-CM-chitosan hydrogel increases due to the transformation of –COOH groups into their deprotonated form (–COO^−^) [13]. This helps to capture the dye molecules using electrostatic attraction between the negatively charged *O*-CM-chitosan hydrogel and positively charged dye.

In contrast, at a low pH, there was an obvious decrease in the adsorption capacity of MB dye onto *O*-CM-chitosan hydrogel. At an acidic medium, the presence of excess H_3_O^+^ ions causes a decrease in the concentration of carboxylate anions (–COO^−^) and an increase in the concentration of the carboxylic groups (–COOH). This reduces the number of negative-charged sites that are needed for the adsorption of cationic dyes. With excess H^+^ ions, the protonation of NH, C=O and C=S groups occurs, which increases the positive charge density on the adsorbent [39]. This hinders the positively charged dye from reaching the *O*-CM-chitosan hydrogel chain, which restricts the adsorption of cationic dyes onto the adsorbent surface. As a result, the electrostatic attraction between the dye molecules and adsorbent surface decreases. This finally results in decreases in the adsorption capacity of *O*-CM-chitosan hydrogel [40].

**Scheme 2:**
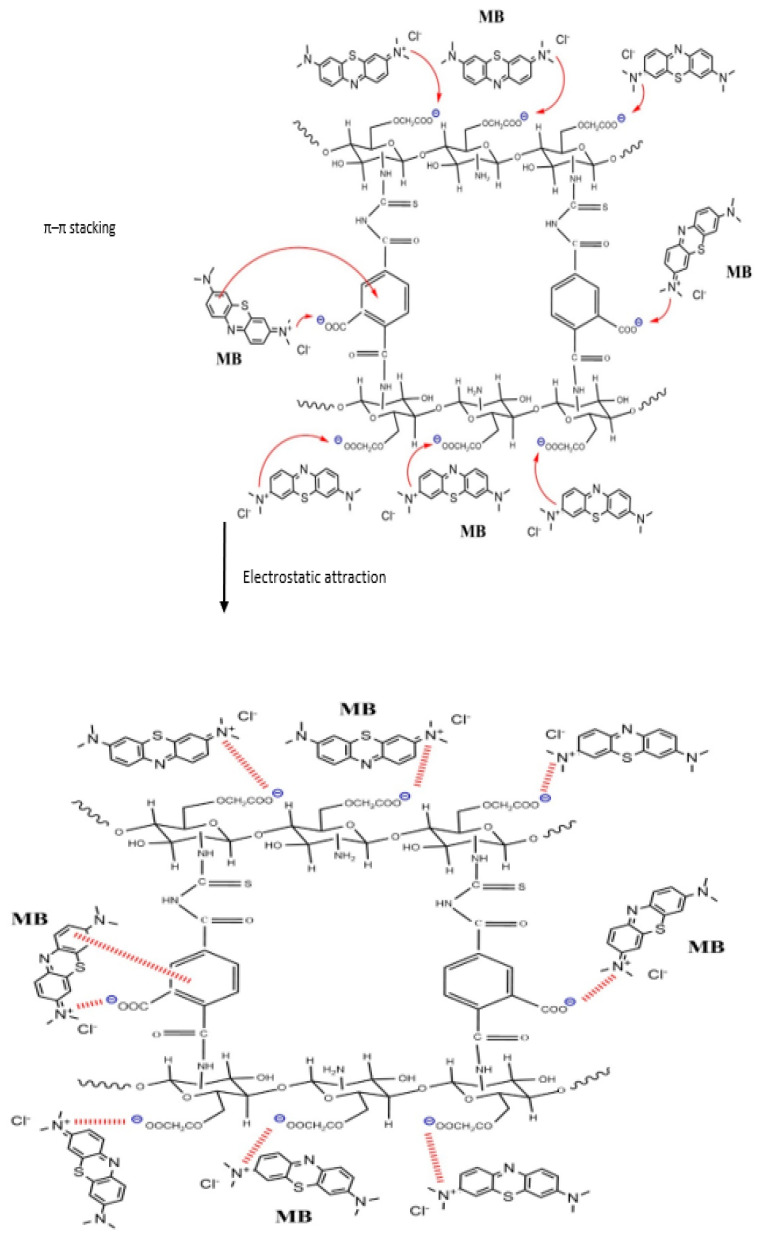
The adsorption mode of MB dye onto the *O*-CM-chitosan hydrogel at pH 9.

The adsorption of MB dye onto *O*-CM-chitosan hydrogel can be attained by intraparticle diffusion and surface adsorption. This can be achieved by various interactions including electrostatic interaction, hydrophobic and π-π stacking. The electrostatic interaction occurs between the negatively charged groups on the adsorbent surface and positively charged groups on dye. The hydrophobic interaction is the association tendency between nonpolar groups of the adsorbent and adsorbate. The π-π stacking interaction is due to the benzene ring on the dye molecule and the benzene ring on the *O*-CM-chitosan hydrogel surface. Therefore, the interaction between *O*-CM-chitosan hydrogel and the dye molecules occurs via electrostatic, hydrophobic and π-π stacking interactions, as shown in Scheme 2 [41].

##### Effect of Initial Dye Concentration

**Figure 8:**
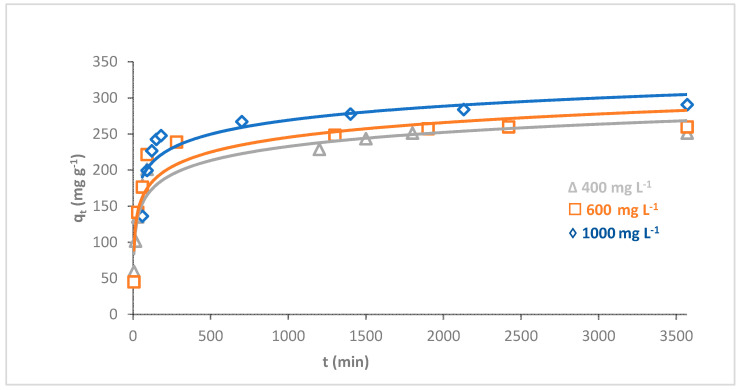
Effect of initial dye concentration on adsorption of MB dye onto *O*-CM-chitosan hydrogel at different time intervals (adsorbent dose 0.05 g, at 25 °C, pH 7 and 50 mL of dye solution with concentration from 400–1000 mg L^−1^).

The influence of the initial dye concentration on the adsorption of MB dye onto *O*-CM-chitosan hydrogel was investigated using a series of different dye concentrations (400, 600 and 1000 mg L^−1^) as a time function (hydrogel amount 0.05 g, 50 mL of dye solution, pH 7 and temperature 25 °C). The adsorption capacities were illustrated in Figure 8.

The results showed a prominent effect of the initial concentration of dye on its adsorption capacity using *O*-CM-chitosan hydrogel. With a constant dose of adsorbent, it is obvious that the removal percentage of dye decreased with increasing initial dye concentration. The removal percentages of MB dye were 62.8, 43.3 and 29.1% at initial dye concentrations of 400, 600 and 1000 mg L^−1^, respectively. On the other hand, the adsorption capacities for dye increased with increments in its initial concentration, from 251.3 to 290.6 mg g^−1^, with increasing initial dye concentration from 400 to 1000 mg L^−1^.

The effect of the initial dye concentration on the amount of adsorption is due to the direct relationship between the sites that are available for adsorption on the adsorbent material and the concentration of the dye. In general, when the initial dye concentration increases, the percentage of dye removal decreases, which may be due to the saturation of the adsorption sites on the surface of the adsorbent [42]. On the other hand, the increase in the initial dye concentration will increase the adsorption capacity, due to the high driving force for mass transfer at a high initial dye concentration [43].

For comparison, the adsorption capacity of MB dye onto the parent non-cross-linked *O*-CM-chitosan (adsorbent dose = 0.05 g, dye solution (50 mL, 1000 mg L^−1^), pH 7 and temperature 25 °C) was found to be 210.1 mg g^−1^, which is lower than that obtained by cross linked *O*-CM-chitosan hydrogel (290.6 mg g^−1^) under the same conditions. Chitosan does not show any adsorption of MB dye. This confirms the role played by cross-linker moieties in the adsorption process.

#### 3.2.2. Kinetic Studies

**Figure 9:**
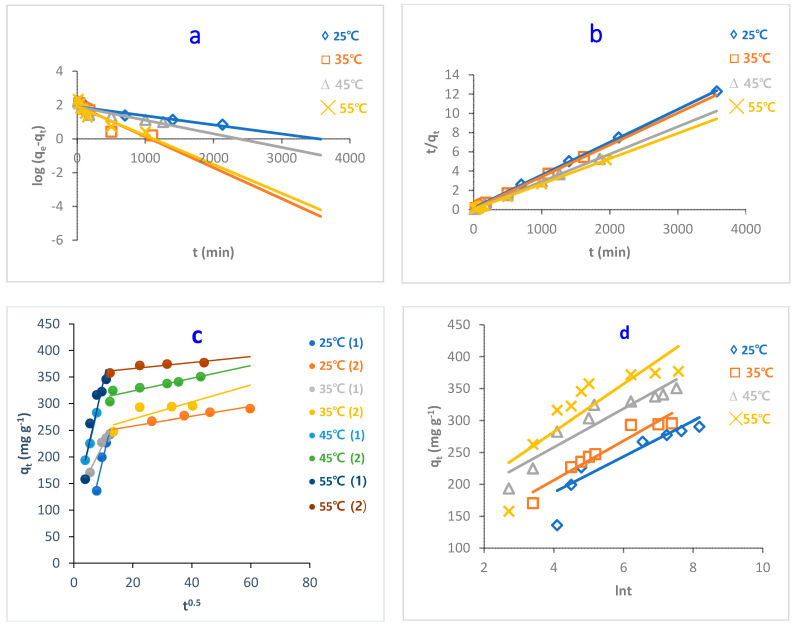
Kinetic models of adsorption of MB dye onto *O*-CM-chitosan hydrogel at different temperatures; (**a**) pseudo-first-order model, (**b**) pseudo-second-order model, (**c**) intraparticle diffusion model and (**d**) Elovich model. Adsorbent mass (0.05 g), dye solution (50 mL, 1000 mg L^−1^), and pH 7.

**Figure 10:**
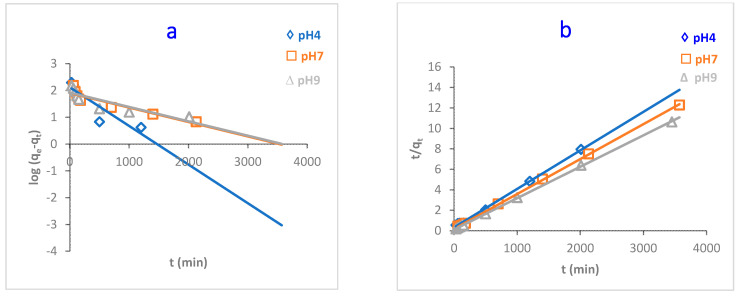

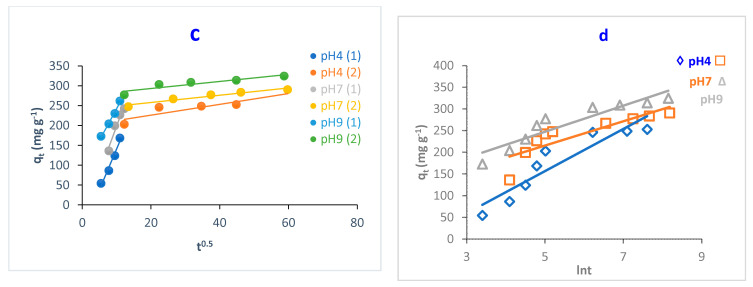
Kinetic models of adsorption of MB dye onto *O*-CM-chitosan hydrogel at different pH; (**a**) pseudo-first-order model, (**b**) pseudo-second-order model, (**c**) intraparticle diffusion model and (**d**) Elovich model. Adsorbent mass (0.05 g), dye solution (50 mL, 1000 mg L^−1^) at 25 °C.

**Figure 11:**
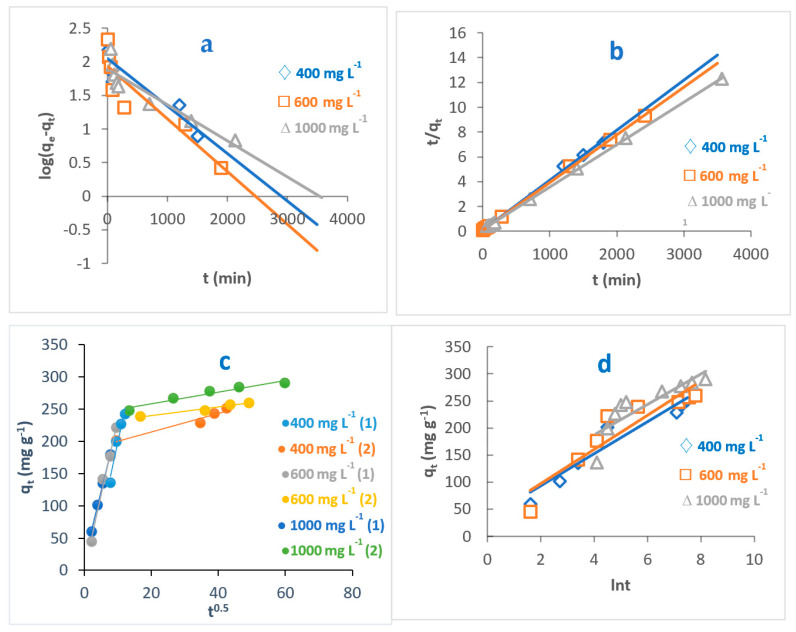
Kinetic models of adsorption of MB dye onto *O*-CM-chitosan hydrogel at different dye concentrations; (**a**) pseudo-first-order model, (**b**) pseudo-second-order model, (**c**) intraparticle diffusion model and (**d**) Elovich model. Adsorbent mass (0.05 g), dye solution (50 mL), temperature 25 °C and pH 7.

The study of adsorption kinetics is very important when evaluating the adsorption efficiency of the adsorbent. This can help to understand and explain the mechanism of the adsorption process. This can help researchers design the adsorption systems [44]. In this regard, to determine the controlling step in the adsorption of MB dye onto *O*-CM-chitosan hydrogel surface, the effect of time on adsorption was studied at different parameters, temperature, pH and dye concentrations, using four kinetic models to fit the experimental data. Pseudo-first-order, pseudo-second-order, intraparticle diffusion and Elovich models (Equations (7)–(10), respectively) were used, as shown in Figure 9, Figure 10 and Figure 11, and the kinetic parameters are summarized in Table 2.

From the results, it can be seen the values of correlation coefficient, R^2^ related to pseudo-second-order models are greater than those obtained from the pseudo-first-order model. This indicates that the adsorption was better described by pseudo-second-order than pseudo-first-order kinetic models, as evidenced by the R^2^ values, which are close to 1. For instance, the R^2^ for values pseudo-second-order models, obtained by the adsorption of MB dye onto *O*-CM-chitosan hydrogel ranged from 0.998 to 1, greater than the range from 0.861 to 0.746 obtained for the pseudo-first-order model.

Furthermore, the calculated values of adsorption capacities, *q_e,cal_*, resulting from the pseudo-second-order model are close to the experimental adsorption capacities, *q_e,exp_*. These were 294.1 and 290.6 mg g^−1^ for calculated and experimental adsorption capacities, respectively. In contrast, corresponding MB values that resulted from the pseudo-first-order model are not close.

Normalized standard deviation Δ*q_e_* (%) was used to compare the pseudo-second order and pseudo-first order kinetic models and determine the best fit between the theoretical values and experimental results for the adsorption of MB dye onto *O*-CM-chitosan. According to the results of Δ*q_e_* (%), given in Table 2, the pseudo-second-order model yielded lower Δ*q_e_* (%) values than those obtained for the pseudo-first-order model, confirming that the pseudo-second-order model is better at describing the adsorption of MB dye onto the *O*-CM-chitosan hydrogel. This finding agreed with the obtained R^2^ values. This implies that the adsorption process is chemisorption, indicating that the rate-limiting step for the adsorption of MB dye onto *O*-CM-chitosan hydrogel involved ion exchange between the adsorbent and adsorbate. This finding is in a good agreement with a recent study for the removal of MB dye using iodate-chitosan assembled composite [45], and the removal of RR 120 dye by cross-linked chitosan-epichlorohydrin bio-beads [46].

On the other hand, the values of rate constant, *k*_2_, increased with increasing temperature and pH. They increased from 6.8 × 10^−5^ to 19.0 × 10^−5^ g mg^−1^ min^−1^ as temperature increased from 25 to 55 °C, respectively. The expanding pore size and diffusion of dye increases from the bulk solution to hydrogel surface. Moreover, the values of rate constant k_2_ decreased from 15.0 × 10^−5^ to 6.8 × 10^−5^ g mg^−1^ min^−1^ as dye concentration increased from 400 to 1000 mg L^−1^. This result agreed well with a study on Remazol Black B removal by Rhizopus arrhizus [47]

Both the pseudo-first-order and pseudo-second-order models are insufficient for identifying the mechanism of dye diffusion because they include all adsorption steps (i.e., external film diffusion, adsorption and intraparticle diffusion). For this reason, the intraparticle diffusion model was proposed, as it can provide the necessary knowledge about the rate-limiting steps. Weber and Morris defined intraparticle diffusion as the rate-controlling factor when the adsorption capacity of a certain adsorbent is a function of square root of time. If the straight line does not pass through the origin, this is considered to demonstrate boundary layer resistance [30].

The *q_t_* versus the square root of time *t*_1/2_ at different parameters (temperature, pH and dye concentration) are illustrated in Figure 9, Figure 10 and Figure 11, and the parameters are listed in Table 2. It is noted that the plot consists of two linear sections with two different slopes. This multilinearity supposes that two or more stages occur during the adsorption process. The resulting two-stage plots were separately evaluated using Equation (9). It was reported that the first-stage linear section was attributed to macropore diffusion; this stage is related to the exterior resistance for transferring the mass surrounding the dye molecules. The second stage was ascribed to the micropore diffusion, which is controlled by intraparticle diffusion [48]. The intercept did not pass across the origin (C ≠ 0), indicating that intraparticle diffusion is not the only step used to determine the adsorption rate of MB dye onto *O*-CM-chitosan hydrogel. Other kinetic processes can also contribute to the completion of adsorption mechanisms [49].

Regarding the values of intraparticle diffusion parameters, *k_p_*_1_ and *k_p_*_2_, obtained from dye adsorption, the adsorption rate of the second stage *k_p_*_2_ was lower than that of the first stage *k*_*p*1_. The first stage is the instantaneous diffusion period *k_p_*_1_, at which the dyes are adsorbed onto the external surface of adsorbent and the most available binding sites are utilized on the *O*-CM-chitosan hydrogel surface. At the second stage, this was saturated on the surface of the adsorbent. This can be attributed to the slow diffusion of MB dye from the surface to the micropores, since fewer accessible binding sites are available for adsorption. The values of *k_p_*_1_ and *k_p_*_2_ were 23.5 and 0.9 mg g^−1^ min^−1/2^ at 25 °C, respectively. Similar results were obtained from the adsorption of cationic Methyl violet and MB dyes onto sepiolite [30].

Consequently, the adsorption of dye onto *O*-CM-chitosan hydrogel is multistep process. Other processes contribute to controlling the rate of the adsorption process, such as bulk diffusion, intraparticle diffusion, the ion exchange process and adsorption–desorption equilibrium [49].

The Elovich model was used on the experimental data using Equation (10); the *q_t_* was plotted versus ln t and the results are illustrated in Figure 9, Figure 10 and Figure 11. α and β values, obtained from the plots, are listed in Table 2. The validity of the Elovich equation proposes that the adsorption process is governed via the chemisorption mechanism. [4]. Additionally, an Elovich model is used to describe the second-order model, under the assumption that the solid surface is energetically heterogeneous [50].

The experimental data for the adsorption of MB dye onto *O*-CM-chitosan hydrogel do not agree with the Elovich model. This is ascribed to the low *R*^2^ values, which ranged between 0.922 and 0.714.

Thus, the pseudo-second-order kinetic model can more efficiently describe the adsorption of MB dye onto *O*-CM-chitosan hydrogel than the other studied models.

#### 3.2.3. Adsorption Isotherm

Adsorption isotherm is important when studying the reactive interactions between the solute and adsorbent. Additionally, it helps to describe various parameters, such as the adsorbing material’s surface, the highest capacity of adsorbing material and the nature of the adsorbate, whether multilayer or monolayer [51].

Adsorption isotherms were studied by applying four models: Langmuir isotherm (Equation (12)), Freundlich isotherm (Equation (15)), Temkin isotherm (Equation (16)) and Dubinin–Radushkevich isotherm (Equation (17)) models. Figure 11 illustrates the fitting of adsorption isotherm models, while Table 3 lists the equilibrium constants and the corresponding fitting correlation coefficients (R^2^).

It is noted that the R^2^ of Langmuir isotherm is close to unity: 0.945. This indicates the high applicability of Langmuir model for the adsorption of MB and dye onto *O*-CM-chitosan hydrogel. Additionally, the maximum monolayer adsorption capacity (q_max_) was 434.8 mg g^−1^, showing great potential for the removal of MB dye using *O*-CM-chitosan hydrogel.

This is in agreement with a previous study on the removal of MB dye using cross-linked chitosan-g-poly (acrylic acid)/bentonite composite [52]. The main assumption of the Langmuir model states that the adsorption process occurs at certain homogeneously identical binding centers on the adsorbent surface. The sorption of adsorbate occurs at this center. Once the dye occupies the binding site, no further adsorption can occur at this site, and dye molecules cannot interact with each other [53]. In this study, based on the Langmuir assumption, the carboxylic groups are homogenously distributed on the hydrogel surface as the surface is homogenous.

Langmuir isotherm is used to predict whether the adsorption process is favorable or unfavorable using the dimensionless constant R_L_. This constant is utilized to determine hydrogel’s affinity towards MB dye (Equation (13)). In the present study, the R_L_ values are 0.2–0.1, confirming the favorability of the adsorption process [22].

On the other hand, the Freundlich isotherm is based on the non-ideal adsorption process: a multilayer adsorption on heterogeneous surfaces [46]. The values of correlation coefficients showed a low linearity of 0.589, confirming the unsuitability of the Freundlich isotherm for adsorption processes.

**Figure 12:**
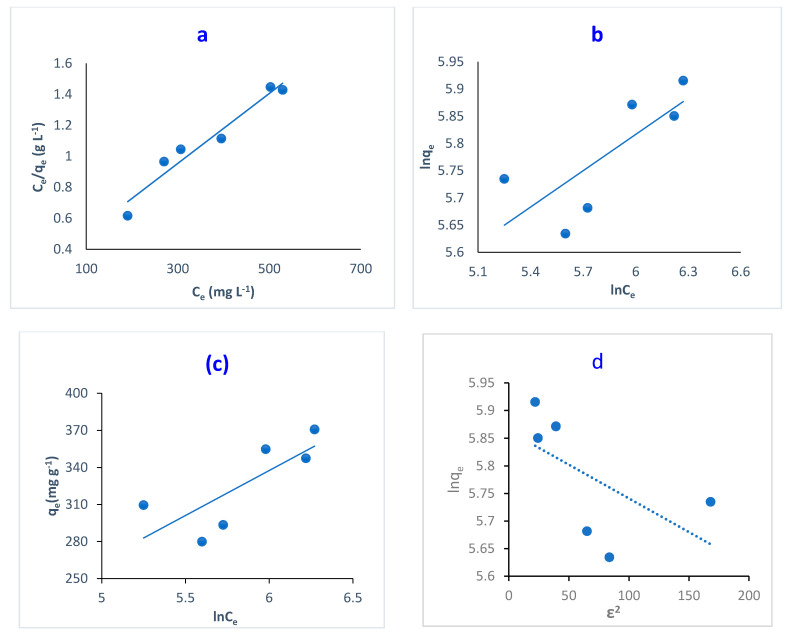
Linear adsorption isotherm for the removal of MB dye by *O*-CM-chitosan hydrogel; (**a**) Langmuir isotherm, (**b**) Freundlich isotherm, (**c**) Temkin isotherm, and (**d**) D-R isotherm models.

The Temkin isotherm model studies the relationship between the heat of adsorption and the surface coverage (Equation (16)). In addition, it assumes an indirect interaction between the adsorbent and adsorbate. Figure 12c illustrated the plot of *q_e_* versus ln *C_e_*. The Temkin parameters B and K_t_ correspond to the adsorption heat and Temkin isotherm equilibrium binding constant, respectively. These were obtained from the gradient and intercept, respectively. The adsorption heat likely decreased by covering as a result of the interaction between the adsorbent and adsorbate [46]. The poor linearity obtained from Figure 12c with low R^2^ values (0.605) revealed the unsuitability of the Temkin model for describing adsorption for MB dye by *O*-CM-chitosan hydrogel surface.

The D-R isotherm is an empirical model, which is used to describe the adsorption mechanism for heterogeneous surfaces. This model is only suitable for intermediate concentrations of adsorbate. Throughout this model, the adsorption process is controlled by a pore-filling mechanism. It is temperature-dependent; therefore, it is mainly used to distinguish between physical and chemical adsorption processes [54].

The linear forms of the D-R isotherm model were illustrated in Figure 12d, showing the adsorption of MB dye onto *O*-CM-chitosan hydrogel. The ln *q_e_* was plotted respective to ε^2^, resulting in a straight line, which was used to determine B and *q_m_* from the slope and intercept, respectively. The D-R parameters are illustrated in Table 3. The low correlation coefficients R^2^ (0.349) confirmed the unsuitability of D-R isotherm for describing the adsorption MB dye.

To analyze the suitability of the four studied models, Figure 12 showed the differences in their fitness for the experimental data. Based on the results, and by comparing the correlation coefficient values of R^2^, illustrated in Table 3, we can conclude that the Langmuir isotherm model showed a better fit for the experimental data on MB adsorption on *O*-CM-chitosan hydrogel than the other isotherm models. This result reflects the homogeneous nature of the *O*-CM-chitosan hydrogel surface [51].

#### 3.2.4. Thermodynamic Studies

**Figure 13:**
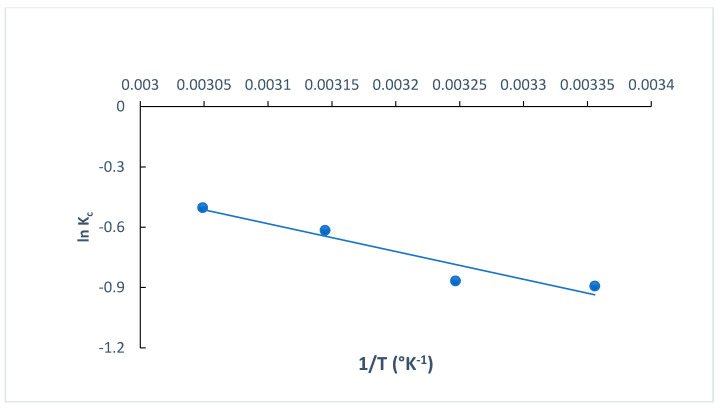
The plots of ln *K_c_* versus 1/*T* for the adsorption of MB dye onto *O*-CM-chitosan hydrogel.

The determination of thermodynamic parameters is crucial when exploring the type of adsorption process, understanding more about the effect of temperature on the adsorption process and identifying whether it is spontaneous or non-spontaneous [35]. The standard change in the free energy (Δ*G°* kJ mol^−1^), standard change in the entropy (Δ*S*° J K^−1^ mol^−1^) and standard change in the enthalpy (Δ*H*° kJ mol^−1^) for the adsorption of MB dye onto *O*-CM-chitosan hydrogel were calculated at different temperatures (298, 308, 318 and 328 K). These parameters are estimated using the van’t Hoff equation (Equations (20) and (21)). The plot of ln K_c_ versus 1/T (Equation (21)) is used to estimate the Δ*H*° and Δ*S*° values from the slope and intercept, respectively. Figure 13 shows the straight lines. Additionally, the thermodynamic parameters are summarized in Table 4.

The Δ*H*° and Δ*S*° values obtained from the adsorption of MB dye onto *O*-CM-chitosan hydrogel were 11.50 kJ mol^−1^ and 30.814 J K^−1^ mol^−1^, respectively, while the Δ*G*° values were 2.21, 2.22, 1.63 and 1.37 KJ mol^−1^ at temperatures of 298, 308, 318 and 328 K, respectively.

The positive enthalpy Δ*H*° value obtained from the adsorption of MB dye confirmed the endothermic nature of the adsorption process [35], revealing the presence of an energy barrier in the adsorption of MB dye onto *O*-CM-chitosan hydrogel. This implied that the dye molecules must displace more than one water molecule before their adsorption on the adsorbent surface [55], indicating the formation of monolayer adsorption [56]. Moreover, the positive entropy values Δ*S*° are attributed to the randomness increment at the interface between the liquid and solid. This showed the high affinity between MB dye molecules and the *O*-CM-chitosan hydrogel surface, and the increased degree of dispersion with rising temperature. This result agreed with the removal of cationic dyes by *N*-benzyl-*O*-carboxymethyl chitosan magnetic nanoparticles [57]. The positive entropy value may be attributed to the randomness increment due to the change in the adsorbent structure, as a result of its interaction and association with MB dye molecules via active groups on the surface [35]. Meanwhile, the adsorption of MB dye onto *O*-CM-chitosan hydrogel is an entropy-driven process [58]. The positive value of Δ*G*° revealed that the process is non-spontaneous. Additionally, Δ*G*° decreased with increasing temperatures.

#### 3.2.5. Activation Energy

**Figure 14:**
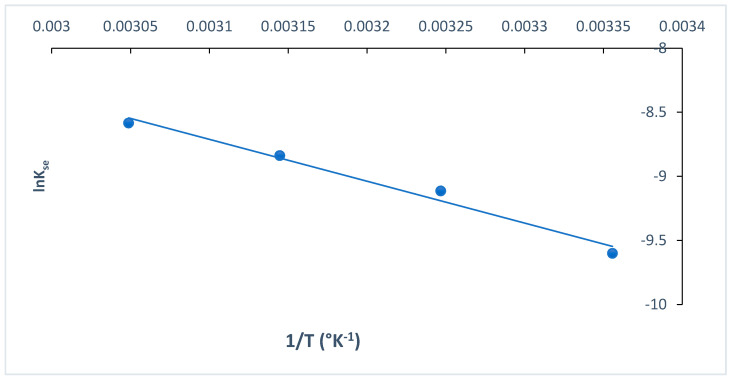
Arrhenius plot for adsorption MB dye onto *O*-CM-chitosan hydrogel.

The rate of MB dye adsorption onto *O*-CM-chitosan hydrogel using Arrhenius equation (Equation (22)) exhibited consistent temperature dependency. The activation energy *E_a_* was obtained as a slope, which resulted by plotting lnk_2_ versus temperature (1/T), as presented in Figure 14.

The activation energy value is used to determine whether the reaction is chemisorption or physisorption. If the activation energy ranged from 5 to 40 kJ mol^−1^, the adsorption corresponded to a physisorption process, while if it ranged from 40 to 800 kJ mol^−1^, the adsorption corresponded to a chemisorption process [35]. In the present study, the *Ea* value was 27.15 kJ mol^−1^. These results indicated the presence of a low potential barrier, corresponding with the physisorption process [52]. Similar results were reported in the literature [55].

### 3.3. Desorption and Regeneration Studies

MB dye was desorbed from O-CM chitosan hydrogel, and the desorption percentage was estimated according to Equation (23). The desorption percentage reached 78%, 63%, 55% and 48% at the first, second, third and fourth cycles, respectively. These results that confirm that the adsorption of MB dye onto O-CM chitosan hydrogel occurs through electrostatic interaction. This finding confirms that the regeneration and reuse of the investigated adsorbent is possible.

## 4. Conclusions

A novel *O*-CM-chitosan hydrogel was prepared by reacting *O*-CM-chitosan with trimellitic anhydride isothiocyanate as a cross-linker. The cross-linking successfully increased the chemical stability of *O*-CM-chitosan hydrogel in acidic medium. Its structure was characterized using different techniques, including FTIR, XRD and SEM analysis. The chemical modification that was achieved by cross-linking resulted in the incorporation of carboxylic groups that act as anionic binding centers for electrostatic interaction with cationic dyes. Its adsorption capacity for Methylene Blue (MB) dye was studied at different temperatures, pH and initial dye concentrations. The MB dye adsorption was fitted to the kinetic model of pseudo-second order, indicating that the adsorption was chemisorption. The intraparticle diffusion models showed multilinearity, confirming that the adsorption of MB dye onto *O*-CM-chitosan hydrogel occurs through different steps. The intercept did not pass through the origin, indicating that intraparticle diffusion was not the only rate-determining step. The isotherms of adsorption of MB dye onto *O*-CM-chitosan hydrogel conform to the Langmuir isotherm model. The obtained maximum monolayer adsorption capacity (q_max_) was 434.8 mg g^−1^. The Δ*H*° (11.50 kJ mol^−1^) values implied that the adsorption of MB dye was an endothermic process. The positive values of Δ*G*° indicated that the adsorption of MB dye was non-spontaneous. The values of the activation energy *E_a_* are 27.15 kJ mol^−1^, indicating that the adsorption for MB dye was physisorption. Based on the resulting data, the adsorption mechanisms for MB dye onto *O*-CM-chitosan hydrogel was physisorption and chemisorption. The regeneration and reuse of the investigated adsorbent is possible as the desorption percentage reached 78% in the first cycle.

## Figures and Tables

**Table 1:** Temperature and pH impact swelling capacity for chitosan, *O*-CM-chitosan, as well as *O*-CM-chitosan hydrogel.

Sample		% Swelling Capacity		
		pH		
	Temperature (°C)	4	7	9
Chitosan	25	847.8	780.7	641.9
	35	857.4	799.1	652.3
	55	884.9	818.2	655.0
*O*-CM-chitosan	25	855.6	869.6	1175.7
	35	1040.0	1102.7	1198.0
	55	1124.0	1190.6	1221.8
*O*-CM-chitosan hydrogel	25	1011.0	1219.7	1317.6
	35	1150.3	1284.6	1415.2
	55	1256.5	1303.5	1705.5

**Table 2:** Kinetic parameters for adsorption of MB dye onto *O*-CM-chitosan hydrogel at different temperatures*,* pH and dye concentrations.

Parameter				Kinetic Models															
			q_e(exp)_	Pseudo-First-Order				Pseudo-Second-Order				Intraparticle Diffusion				Elovich Model			Removal Efficiency %
Temp.	pH	Conc.		*q*_1_	*R*_1_	*k*_1_ (10^−5^)	Δ *q_e_*%	*q*_2_	*R*_2_	*K*_2_ (10^−5^)	Δ *q_e_*%	*k_p_*_1_	*R*_2_ *P*_1_	*k*_p2_	*R*_2_ *P*_2_	β	ἀ	*R*_2_	
**25 °C**	**7**	**1000**	290.6	79.2	0.861	0.001	25.7	294.1	0.999	6.8	0.4	23.5	0.944	0.9	0.948	0.036	424	0.744	29.1
**35 °C**	**7**	**1000**	295.8	91	0.854	0.004	26.2	303	0.999	11	0.9	11.1	0.963	1.6	0.656	0.032	445	0.923	29.6
**45 °C**	**7**	**1000**	350.8	79.9	0.768	0.002	27.3	344.8	0.999	14	0.6	23.1	0.995	1.2	0.841	0.033	2859	0.877	35.1
**55 °C**	**7**	**1000**	377	79.4	0.803	0.004	27.9	384.6	1	19	0.7	24	0.847	0.6	0.796	0.027	1340	0.714	37.7
**25 °C**	**4**	**1000**	252.9	130.3	0.813	0.003	17.1	263.2	0.998	4.5	1.6	20.6	0.971	1.4	0.696	0.021	8	0.861	25.3
**25 °C**	**7**	**1000**	290.6	79.2	0.861	0.001	25.7	294.1	0.999	6.8	0.4	23.5	0.944	0.9	0.948	0.036	424	0.744	29.1
**25 °C**	**9**	**1000**	324.6	83.4	0.746	0.001	26.3	322.6	0.999	8.6	0.2	16	0.99	0.9	0.849	0.033	747	0.864	32.5
**25 °C**	**7**	**400**	251.3	113.6	0.856	0.002	20.7	250	0.998	15	0.2	21.5	0.996	1.4	0.949	0.034	90	0.922	62.8
**25 °C**	**7**	**600**	259.7	87.5	0.828	0.002	25.1	263.2	0.999	14	0.5	23.7	0.985	0.7	0.965	0.032	94	0.864	43.3
**25 °C**	**7**	**1000**	290.6	79.2	0.861	0.001	25.7	294.1	0.999	6.8	0.4	23.5	0.944	0.9	0.948	0.036	424	0.744	29.1

Conc.: mg L^−1^; *q_e(exp)_*, *q*_1,_ *q*_2:_ mg g^−1^; *k*_1_: g mg^−1^min^−1^; *k*_2_: g mg^−1^min^−1^; *k*_*p*1_, *k*_*p*2:_ mg g^−1^ min^−1∕2^; β: g mg^−1^; ἀ: mg g^−1^ min^−1.^

**Table 3:** Adsorption isotherm parameters for removal of MB dye by *O*-CM-chitosan hydrogel.

Models	Parameter	
**Langmuir**	q_max_ (mg g^−1^)	434.782
	*R_L_*	(0.2–0.1)
	*K*_*L*_ (L mg^−1^)	0.008
	*R* ^2^	0.945
**Freundlich**	1/*n*	0.2
	*K_f_* (mg g^−1^)	88.5
	*R* ^2^	0.589
**Temkin**	*B* (kJ mol^−1^)	72.9
	*K_T_* (L g^−1^)	3.7
	*R* ^2^	0.605
**D-R**	*q_m_* (mg g^−1^)	315.8
	EkJ mol^−1^	0.02
	*B*	0.001
	*R* ^2^	0.349

**Table 4:** Thermodynamic data for the adsorption of MB dye by *O*-CM-chitosan hydrogel at different temperatures.

Temp. (K)	Δ*G*° (KJ mol^−1^)	Δ*H*° (kJ mol^−1^)	Δ*S*° (J K^−1^ mol^−1^)	*E*_*a*_ (kJ mol^−1^)
298	2.21	11.5	30.81	27.15
308	2.22			
318	1.63			
328	1.37			

## Data Availability

The data presented in this study are available on request from the corresponding author.
